# Impacts of NaHCO_3_ on β-Lactam Binding to PBP2a Protein Variants Associated with the NaHCO_3_-Responsive versus NaHCO_3_-Non-Responsive Phenotypes

**DOI:** 10.3390/antibiotics11040462

**Published:** 2022-03-30

**Authors:** Selvi C. Ersoy, Liana C. Chan, Michael R. Yeaman, Henry F. Chambers, Richard A. Proctor, Kevin C. Ludwig, Tanja Schneider, Adhar C. Manna, Ambrose Cheung, Arnold S. Bayer

**Affiliations:** 1 The Lundquist Institute for Biomedical Innovation, Harbor-UCLA Medical Center, Torrance, CA 90502, USA; selvi.ersoy@lundquist.org (S.C.E.); lchan@lundquist.org (L.C.C.); mryeaman@ucla.edu (M.R.Y.); 2 David Geffen School of Medicine, University of California, Los Angeles, CA 90095, USA; 3 Division of Infectious Diseases, Department of Medicine, Harbor-UCLA Medical Center, Torrance, CA 90502, USA; 4 Division of Molecular Medicine, Department of Medicine, Harbor-UCLA Medical Center, Torrance, CA 90502, USA; 5 School of Medicine, University of California-San Francisco (UCSF), San Francisco, CA 94143, USA; henry.chambers@ucsf.edu; 6 Departments of Medicine and Medical Microbiology/Immunology, School of Medicine and Public Health, University of Wisconsin, Madison, WI 53715, USA; rap@wisc.edu; 7 Institute for Pharmaceutical Microbiology, University Hospital Bonn, University of Bonn, D-53113 Bonn, Germany; kevin.ludwig@uni-bonn.de (K.C.L.); tschneider@uni-bonn.de (T.S.); 8 Department of Microbiology & Immunology, Geisel School of Medicine at Dartmouth, Hanover, NH 03755, USA; adhar.c.manna@dartmouth.edu (A.C.M.); ambrose.cheung@dartmouth.edu (A.C.)

**Keywords:** penicillin-binding proteins, sodium bicarbonate, β-lactams, methicillin-resistant *Staphylococcus aureus*

## Abstract

Methicillin-resistant *Staphylococcus aureus* (MRSA) regulates resistance to β-lactams via preferential production of an alternative penicillin-binding protein (PBP), PBP2a. PBP2a binds many β-lactam antibiotics with less affinity than PBPs which are predominant in methicillin-susceptible (MSSA) strains. A novel, rather frequent in vitro phenotype was recently identified among clinical MRSA bloodstream isolates, termed “NaHCO_3_-responsiveness”. This phenotype features β-lactam susceptibility of certain MRSA strains only in the presence of NaHCO_3_. Two distinct PBP2a variants, 246G and 246E, have been linked to the NaHCO_3_-responsive and NaHCO_3_-non-responsive MRSA phenotypes, respectively. To determine the mechanistic impact of PBP2a variants on β-lactam susceptibility, binding profiles of a fluorescent penicillin probe (Bocillin-FL) to each purified PBP2a variant were assessed and compared to whole-cell binding profiles characterized by flow cytometry in the presence vs. absence of NaHCO_3_. These investigations revealed that NaHCO_3_ differentially influenced the binding of the fluorescent penicillin, Bocillin-FL, to the PBP2a variants, with binding intensity and rate of binding significantly enhanced in the 246G compared to the 246E variant. Of note, the NaHCO_3_-β-lactam (oxacillin)-responsive JE2 strain, which natively harbors the 246G variant, had enhanced Bocillin-FL whole-cell binding following exposure to NaHCO_3_. This NaHCO_3_-mediated increase in whole-cell Bocillin-FL binding was not observed in the NaHCO_3_-non-responsive parental strain, COL, which contains the 246E PBP2a variant. Surprisingly, genetic swaps of the *mecA* coding sites between JE2 and COL did not alter the NaHCO_3_-enhanced binding seen in JE2 vs. COL. These data suggest that the non-coding regions of *mecA* may be involved in NaHCO_3_-responsiveness. This investigation also provides strong evidence that the NaHCO_3_-responsive phenotype in MRSA may involve NaHCO_3_-mediated increases in both initial cell surface β-lactam binding, as well as ultimate PBP2a binding of β-lactams.

## 1. Introduction

The methicillin-resistant *Staphylococcus aureus* (MRSA) phenotype is a major obstacle to the effective treatment of severe *S. aureus* clinical infections, including bacteremia and infective endocarditis [1]. MRSA strains are typically resistant to nearly all β-lactams (first-line treatment of choice for methicillin-susceptible *S. aureus* (MSSA)), on standard in vitro testing, linked to their preferential production of an alternative penicillin-binding protein (PBP), PBP2a [2,3]. Most β-lactam antibiotics bind poorly to PBP2a when tested in standard microbiologic growth media, allowing cell wall synthesis and microbial proliferation to occur in their presence [4,5].

Recently, a novel phenotype was identified amongst clinical MRSA strains, whereby certain isolates exhibit susceptibility to early generation β-lactams (e.g., cefazolin and/or oxacillin) in the presence of NaHCO_3_ supplementation of standard media in vitro [6,7,8,9,10]. Physiological relevance of this phenomenon in terms of β-lactam re-sensitization has been verified in both ex vivo and in vivo models [6,11]. This β-lactam hypersusceptibility appears to be linked, at least in part, to NaHCO_3_-mediated repression of the *mecA*/*blaZ*/PBP2a axis, as well as alteration in several accessory genes required for proper PBP2a functionality and maturation, ultimately impacting cell wall synthesis [6,9,12]. However, the precise effects of NaHCO_3_ on specific properties of the PBP2a protein itself are currently unknown.

Of interest, another novel β-lactam hypersusceptibility phenotype was recently identified in MRSA, wherein strains with particular *mecA* genotypes were rendered susceptible to the combination of β-lactams and β-lactamase inhibitors [13]. Importantly, two particular PBP2a protein variants, 246E (wild-type PBP2a [13]) and 246G, were observed to have differing binding affinities for penicillin in the presence of clavulanic acid [13]. This difference was hypothesized to contribute to altered susceptibilities to penicillin + clavulanic acid in strains harboring these variants. 

In the current study, we queried whether such specific PBP2a variants could also influence the NaHCO_3_-responsive/non-responsive phenotypes in a parallel manner. Furthermore, we aimed to determine whether NaHCO_3_ affected PBP2a-β-lactam binding affinities and temporal binding profiles, in a genotype-specific process (similar to penicillin + clavulanate). 

Herein, purified PBP2a variants 246G and 246E, prototypic of the NaHCO_3_-responsive and -non-responsive phenotypes, respectively [10], were compared for their binding affinities for the fluorescently labeled penicillin derivative, Bocillin-FL, in the presence and absence of NaHCO_3_. Furthermore, responsive and non-responsive strain sets harboring either their native or genetically swapped PBP2a variants (246G and 246E) were also assessed for ability to bind Bocillin-FL in the presence vs. absence of NaHCO_3_ by whole-cell flow cytometry. 

## 2. Results

### 2.1. Bocillin-FL Binding to Purified PBP2a

**Figure 1:**
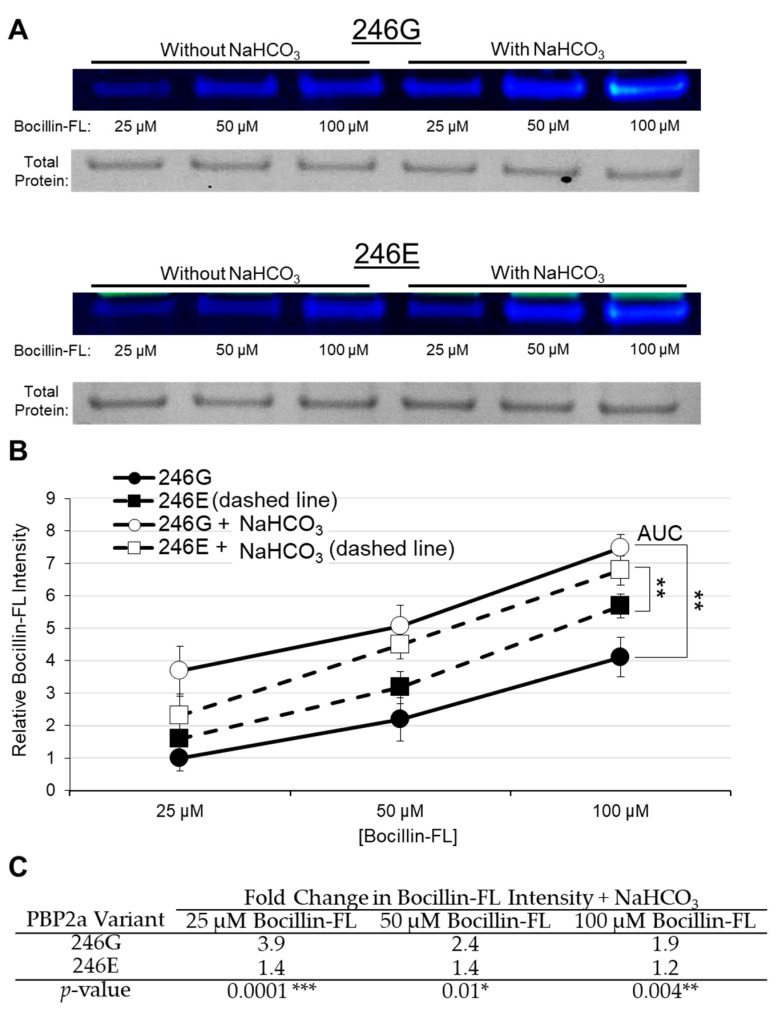
Bocillin-FL binding to purified PBP2a variants 246G and 246E. (**A**) Representative gel images for PBP2a-Bocillin binding and total protein loading; 50 µg/mL PBP2a was incubated with 25, 50, or 100 µM Bocillin-FL in each gel. (**B**) Bocillin-FL binding with and without 44 mM NaHCO_3_; 50 µg/mL PBP2a was used in each Bocillin binding reaction. Relative Bocillin intensity was determined by normalizing absolute intensity values for each sample to the average intensity of 25 µM Bocillin bound to 50 µg/mL 246G PBP2a in the absence of NaHCO_3_ (with this value being set equal to 1.0). Additionally, an intra-gel normalization control was run on each gel consisting of 25 µM Bocillin-FL incubated with 50 µg/mL 246G PBP2a to which all bands were initially normalized. The data presented are the composite of four separate gel runs. Asterisks represent the significant difference in the area under the curve (AUC) for each variant incubated with and without NaHCO_3_ (Student’s *t*-test, ** *p* < 0.01). (**C**) Fold changes in Bocillin-FL binding to each PBP2a variant in the presence and absence of 44 mM NaHCO_3_ (calculated from data in part (**B**)). Fold change is calculated as [Bocillin Intensity]_NaHCO3_/[Bocillin Intensity]_No NaHCO3_. Values greater than 1 indicate increased Bocillin-FL binding to PBP2a in the presence of NaHCO_3_. Statistical significance is calculated using the average fold-change in Bocillin-FL binding for each PBP2a variant in the presence vs. absence of NaHCO_3_ by Student’s *t*-test (* *p* < 0.05, ** *p* < 0.01, *** *p* < 0.001).

To study penicillin binding in vitro, *mecA* genes of *S. aureus* COL and USA300 JE2, encoding for PBP2 variants 246E and 246G, respectively, were cloned into expression vectors for heterologous overexpression and Ni-NTA purification. Purified PBP2a proteins of each variant type were utilized in a Bocillin-FL binding assay to determine whether alteration at the 246th amino acid position impacts direct binding of Bocillin-FL to either purified variant. In the absence of NaHCO_3_ in the binding reaction, both variants bound similar amounts of Bocillin-FL (Figure 1A,B). Corroborating this result, in a β-lactam competition assay with either “cold” penicillin or “cold” oxacillin, both variants had similar IC_50_ values for these two antibiotics (Supplementary Materials Figure S1A,B). These findings correspond to those observed by Harrison et al. [13], wherein all PBP2a variants studied had similar affinities for β-lactams under standard assay conditions. 

**Figure 2:**
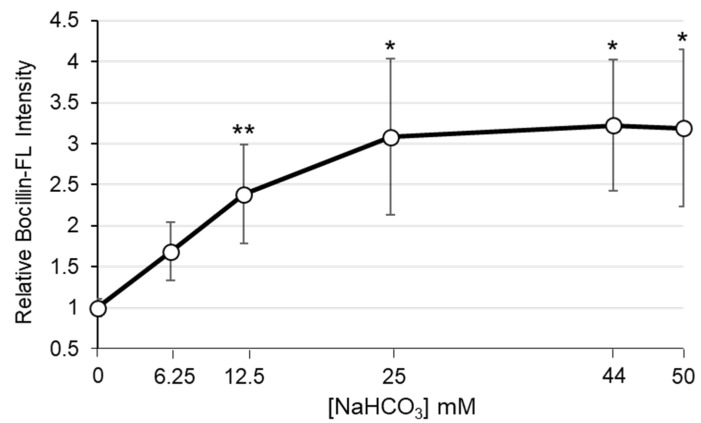
NaHCO_3_ dose response impact on Bocillin-FL binding to purified PBP2a variant 246G; 50 µg/mL PBP2a was incubated with 50 µM Bocillin-FL and either 0, 6.25, 12.5, 25, 44, or 50 mM NaHCO_3_. Relative Bocillin intensity was normalized to a control lane consisting of 25 µM Bocillin-FL incubated with 50 µg/mL 246G PBP2a run on each gel. Final data was normalized to the intensity of 50 µg/mL PBP2a incubated with 50 µM Bocillin-FL and no NaHCO_3_ (with this value set equal to 1.0). Asterisks represent significant differences in Bocillin-FL intensity at the indicated NaHCO_3_ concentration as compared to no NaHCO_3_ (Student’s *t*-test, * *p* < 0.05, ** *p* < 0.01).

Next, in parallel studies, NaHCO_3_ (44 mM) was added to each of the PBP2a-Bocillin-FL binding reactions. This concentration of NaHCO_3_ is the standard used in prior in vitro and ex vivo assays to disclose β-lactam susceptibility and, thus, identify NaHCO_3_-responsive strains [6,7,8,10,11]. When NaHCO_3_ was added to the binding reactions, Bocillin-FL binding to both PBP2a variants was substantially enhanced (Figure 1A,B). However, the binding enhancement to the 246G variant was significantly greater than that observed with the 246E variant (Figure 1C). The impact of NaHCO_3_ on Bocillin-FL binding to the 246G variant was most evident at 25 µM Bocillin-FL, in which NaHCO_3_ stimulated a nearly 4-fold increase in Bocillin-FL intensity, compared to only a 1.4-fold change in intensity in the 246E variant (Figure 1C). Furthermore, to examine the NaHCO_3_ concentration-dependent impact on binding to purified PBP2a, we exposed 50 µg/mL of the 246G variant to 50 µM Bocillin with increasing concentrations of NaHCO_3_ ranging from 0 to 50 mM. There was a significant increase in Bocillin binding at the lower NaHCO_3_ concentrations (** *p* = 0.007 [Student’s *t*-test] comparing 0 mM vs. 12.5 mM), with a more gradual increase in binding between 12.5 and 50 mM (Figure 2).

**Figure 3:**
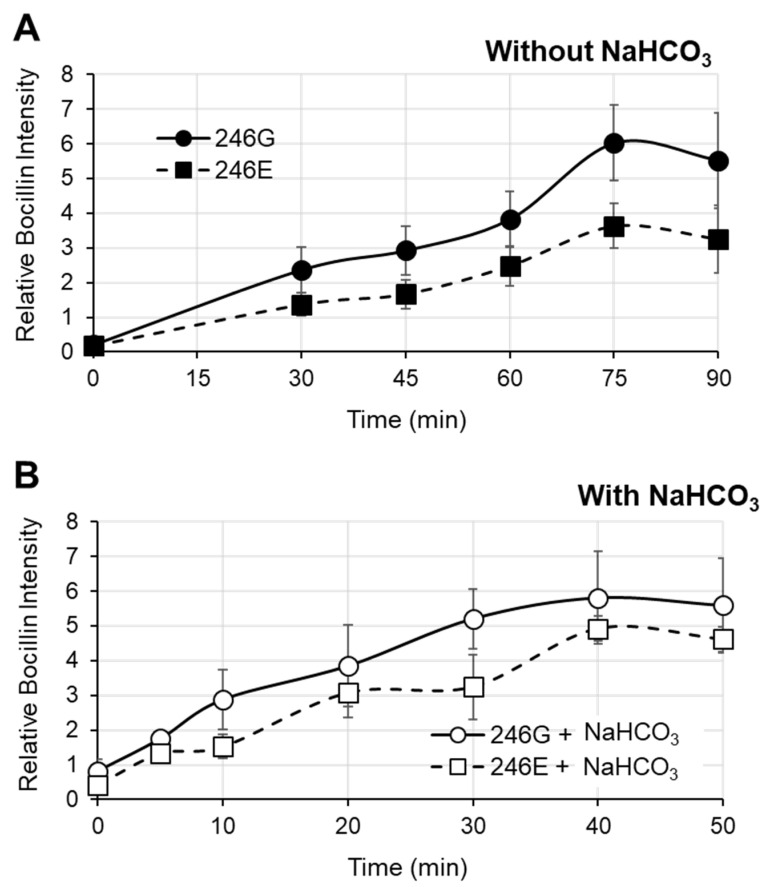
Kinetic binding of Bocillin-FL to purified PBP2a variants 246G and 246E. (**A**) Kinetic Bocillin-FL binding without NaHCO_3_. (**B**) Kinetic Bocillin-FL binding with NaHCO_3_. A total of 50 µg/mL PBP2a was used in each Bocillin binding reaction. Relative Bocillin intensity was determined by normalizing absolute intensity values for each sample to the intensity of 25 µM Bocillin bound to 50 µg/mL 246G PBP2a in the absence of NaHCO_3_ incubated for 20 min run on the same gel.

To investigate the differential impact of NaHCO_3_ on the kinetics of Bocillin-FL binding to each PBP2a variant, 246G PBP2a and 246E PBP2a were incubated with or without NaHCO_3_ and assessed for binding at various time points (Figure 3A,B). These data query time points were established as optimal for this experimentation based on extensive pilot studies. Based on the binding curves, the time to reach 50% maximal binding was calculated for each variant in the presence vs. absence of NaHCO_3_. In the absence of NaHCO_3_, both variants exhibited similar times to reach 50% maximal binding (Figure 3A; 246G 50% binding time = 44.2 ± 4.6 min, 246E 50% binding time = 40.3 ± 8.8 min, Student’s *t*-test *p* = NS). However, in the presence of NaHCO_3_, the 246G variant reached 50% maximal binding significantly faster than the 246E variant (Figure 3B; 246G—50% binding time = 7.6 ± 1.1 min, 246E—50% binding time = 13.2 ± 4.8 min, Student’s *t*-test * *p* = 0.04). For both variants, NaHCO_3_ significantly decreased the time until 50% maximal binding vs. in the absence of NaHCO_3_ (246G without vs. with ** *p* = 0.006; 246E without vs. with * *p* = 0.03).

### 2.2. Bocillin-FL Binding to Whole JE2 and COL Cells by Flow Cytometry

To determine the impact of NaHCO_3_ on binding of Bocillin-FL to whole cells, we compared two well-characterized MRSA strains: JE2 (NaHCO_3_-responsive (Supplementary Materials Table S1); 246G PBP2a variant); vs. COL (-non-responsive (Table S1); 246E PBP2a variant). Neither strain produces β-lactamase [14]. Cells were incubated with 100 µM Bocillin-FL, and then assessed for overall binding by flow cytometry. Prior to staining, cells were grown to mid-log phase (OD_600 nm_ = 0.5) in CA-MHB Tris media plus half the minimum inhibitory concentration (MIC) of oxacillin (Table S1), with or without 44 mM NaHCO_3_.

**Figure 4:**
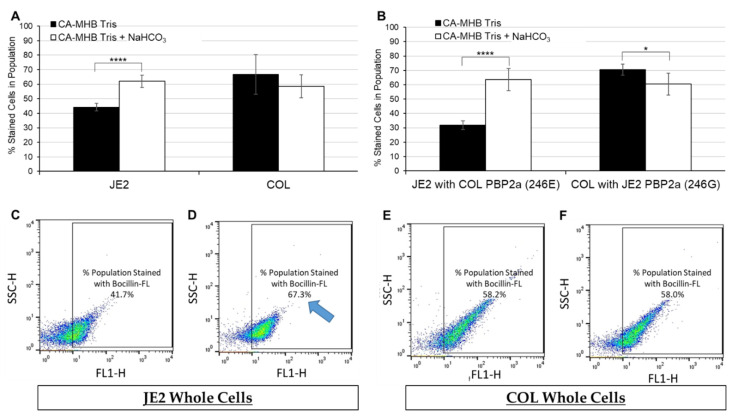
Percentage of JE2 and COL cells out of total population (10,000 cells) stained with Bocillin-FL following growth in cation-adjusted Mueller Hinton broth (CA-MHB Tris) ± 44 mM NaHCO_3_. (**A**) Summary of average Bocillin-FL binding for JE2 and COL. (**B**) Summary of average Bocillin-FL binding for JE2 and COL strains with “swapped” *mecA* coding regions. (**C**–**F**) Representative dot-plots for (**C**) JE2 grown in CA-MHB Tris (**D**) + NaHCO_3_, vs. COL grown in (**E**) CA-MHB Tris (**F**) + NaHCO_3_. Gates on dot-plots (box surrounding cells) depict percentage of cells in total population of 10,000 cells that have taken up the Bocillin-FL dye. Note enhanced proportion of JE2 cells stained with Bocillin-FL in the presence vs. absence of NaHCO_3_ (blue arrow). Statistics were calculated by a Student’s *t*-test, * *p* < 0.05, **** *p* < 0.0001.

Upon analysis, JE2 cells grown in the presence of NaHCO_3_ displayed a significantly greater number of Bocillin-FL-stained cells within the population vs. cells grown in the absence of NaHCO_3_ (Figure 4A,C,D). This NaHCO_3_-induced enhancement of Bocillin-FL binding to JE2 cells corresponds to the NaHCO_3_-stimulated β-lactam sensitization observed in this strain by MIC testing (Table S1). Growth in the presence of NaHCO_3_ had no impact on Bocillin-FL binding to the -non-responsive strain, COL (Figure 4A,E,F), corresponding to the lack of impact of NaHCO_3_ on β-lactam susceptibility by MIC for this strain (Table S1). 

To further investigate the specific impact of NaHCO_3_ on Bocillin-FL whole-cell binding in the presence of each PBP2a variant within a given strain background, “swap” mutants were constructed in JE2 and COL so that each strain possessed the *mecA* coding region of the other strain (JE2 with *mecA* 246E, COL with *mecA* 246G). As expected, swap of only the *mecA* coding regions did not alter the NaHCO_3_ responsiveness of these variants to oxacillin by MIC testing (Table S1). This relates to the requirement of both the *mecA* coding regions and ribosomal-binding sites (RBS) for full expression of this phenotype (Ersoy et al.; manuscript submitted—in review *Antimicrobial Agents and Chemotherapy*).

Interestingly, upon flow analysis of these mutant constructs in the presence vs. absence of NaHCO_3_, each “swap” strain derivative displayed the same phenotype as its parental strain (Figure 4B and Figure S2). This indicates that the NaHCO_3_ enhancement of Bocillin-FL binding to JE2 whole cells is not due solely to increased affinity of this particular PBP2a variant for Bocillin-FL in the presence of NaHCO_3_. This outcome may implicate a likely role of differences in NaHCO_3_ impacts on selected cell surface properties specific to each strain background.

In parallel, the mean fluorescence intensity (MFI) of the cell population was assessed in the presence or absence of NaHCO_3_ exposures. Despite the significant enhancement of the proportion of JE2 (vs. COL) cells binding Bocillin-FL in the presence of NaHCO_3_, the amount of fluorophore taken up per cell population (10,000 cells) did not differ between cells grown in the presence vs. absence of NaHCO_3_ for JE2 vs. COL parental strains, nor COL possessing JE2 PBP2a (Figure S3). 

## 3. Discussion

Investigations with purified PBP2a variants, as well as whole cells harboring differing PBP2a variants, revealed that NaHCO_3_ substantially and differentially influenced the ability of a β-lactam probe, Bocillin-FL, to bind each PBP2a variant. The purified 246G PBP2a variant (previously associated with NaHCO_3_-responsiveness [10]) displayed enhanced binding of Bocillin-FL when NaHCO_3_ was incorporated into the reaction mixture (vs. the 246E variant) in a NaHCO_3_ dose-dependent manner. Prior in vitro data demonstrated that in NaHCO_3_-responsive MRSA harboring the 246G PBP2a variant, β-lactam MICs also decreased in a NaHCO_3_ concentration-dependent-manner. For example, oxacillin and cefazolin MICs for NaHCO_3_-responsive strain MRSA 11/11 were each 4 µg/mL at 25 mM of NaHCO_3_, decreasing 8-fold to 0.5 µg/mL at 44 mM NaHCO_3_ [6].

In parallel to the binding studies with purified PBP2a, whole-cell binding assays demonstrated that exposure to NaHCO_3_ during the early to mid-log growth phases resulted in enhanced Bocillin-FL binding to the NaHCO_3_-responsive strain, JE2, harboring the 246G variant vs. COL, harboring the 246E variant. These NaHCO_3_-stimulated increases in Bocillin-FL whole-cell binding again correspond to NaHCO_3_ impacts on β-lactam susceptibility by MIC testing in vitro for each strain harboring these PBP2a variants (as detailed above). Interestingly, further in-parallel studies utilizing JE2 and COL mutant strains with “swapped” PBP2a variants (JE2 now with 246E vs. COL now with 246G) displayed similar whole-cell binding affinities in the presence vs. absence of NaHCO_3_ as their parental counterparts. These data suggest the possibility that, in addition to NaHCO_3_-mediated enhancements of Bocillin-FL binding to the 246G PBP2a variant, NaHCO_3_ may also be differentially affecting specific surface factors which influence overall Bocillin-FL binding to whole cells. In particular, we have previously shown that NaHCO_3_ can specifically repress transcription of the *prsA* gene and PrsA protein production in NaHCO_3_-responsive but not NaHCO_3_-non-responsive MRSA [9]. The *prsA* gene encodes the chaperone PrsA; this protein is important for the optimal translocation, folding, and maturation of PBP2a [15,16]. In addition to its chaperone function, PrsA is known to regulate the expression of a multitude of surface proteins and exoproteins. Moreover, alteration of PrsA expression can result in a variety of other phenotypic alterations to the cell surface, including perturbations of lipid composition and localization, which can also impact surface protein distribution and function [17,18]. Thus, NaHCO_3_-mediated impacts on *prsA* could plausibly impact net whole-cell binding of Bocillin-FL. In addition to NaHCO_3_ impacts on PrsA, NaHCO_3_ is known to differentially affect the expression of a variety of surface-associated genes/proteins, including the *cap* operon, *pbp2*, and *fmtA*, among others, in NaHCO_3_-responsive vs. non-responsive strain backgrounds [12].

As reported previously [13], both the 246G and 246E PBP2a variants had similar intrinsic binding affinities for Bocillin-FL, as well as to the β-lactams, penicillin, and oxacillin, in the absence of NaHCO_3_. This indicates that factors in the extracellular environment during NaHCO_3_ exposure likely play a crucial role in dictating binding affinities of such fluorophores, as would generally be expected for biochemical interactions. It should be noted that the precise surface factors (e.g., cell wall proteins or membrane lipids) that Bocillin-FL initially binds to in whole cells are not known. 

The profound genetic regulatory effects of NaHCO_3_ on PBP2a expression and function have been recently detailed [9]. However, the precise and direct physio-chemical impacts of NaHCO_3_ on the PBP2a molecule itself following whole-cell binding and intracellular transport are unknown [19]. Since NaHCO_3_ is a weak base, it is likely that pH effects may impact certain aspects of tertiary folding of the PBP2a protein, such as hydrogen bonding and the formation of salt bridges [20,21]. Furthermore, alterations to environmental pH and temperature are known to have catastrophic effects on PBP2a protein folding and functionality [22]. It is unclear whether alterations in Bocillin-FL-PBP2a binding stimulated by NaHCO_3_ are specific to the bicarbonate molecule or are a more generalized weak acid–base effect. Further studies with other weak acids and bases are needed to verify if these impacts are NaHCO_3_-specific or related to more generalized impacts on PBP2a protein structure. In this regard, previous studies by us and others suggest that the “NaHCO_3_ effect” microbiologically is not seen with other weak acids (e.g., salicylic acid; boric acid [6,23]).

Regulation of PBP2a protein activity occurs via allosteric binding interactions, wherein peptidoglycan precursors bind to the allosteric site, causing a conformational change that reveals the active site and allows peptidoglycan synthesis to progress [20,24,25]. Traditional β-lactams (with the exception of ceftaroline) are unable to efficiently occupy this allosteric binding site, allowing the active site to remain closed unless peptidoglycan precursor substrates are present [24,25,26,27]. Although it is difficult to determine the specific impact of NaHCO_3_ on PBP2a protein structure, it is possible that this weak base may alter the allosteric site in a manner that allows better β-lactam binding to both the allosteric and subsequently to the active sites (e.g., as seen in the 246G variant). Indeed, the position of 246th amino acid residue is centrally located within the PBP2a allosteric site [20]. As the 246E variant is considered the “wild type” form of PBP2a [13], it is plausible that the glutamic acid at this residue aids in stabilization of the allosteric binding site compared to variants possessing a glycine at this residue. Thus, it could be hypothesized that the weak acid nature of glutamic acid buffers NaHCO_3_ better than glycine, allowing more stable protein interactions.

Overall, these data support the notion that NaHCO_3_ differentially enhances the binding affinities of selected β-lactams to both the whole cell surface, as well as to the 246G PBP2a variant. This event may, in turn, contribute to the NaHCO_3_-responsive phenotype observed in strains possessing these particular protein variants. Our current studies with mutant strains harboring each of these *mecA* alleles yielded additional mechanistic insights concerning specific PBP2a protein variants and the NaHCO_3_-responsive/-non-responsive phenotypes. Furthermore, since the *mecA* variants we have studied also differ in their ribosomal-binding site (RBS) sequences [10,13], the specific impacts of these latter differences on NaHCO_3_-responsiveness remain to be determined. We are currently examining this concept by transgenic (“swapping”) methods similar to the present investigation, targeting both the *mecA* coding and/or RBS regions between the same NaHCO_3_-responsive/-non-responsive strains used currently. Moreover, we recognize that the data presented here are only comparing β-lactam binding metrics in a single strain set, and need to be validated in additional MRSA strains featuring these two *mecA* variants. Finally, the impact of these two PBP2a variants on binding profiles to other prominent PBPs (e.g., PBP2) needs to be adjudicated. Such studies are in progress.

## 4. Materials and Methods

### 4.1. Bacterial Strains and Growth Conditions 

The NaHCO_3_-responsive strain, JE2 (PBP2a 246G variant), and -non-responsive strain, COL (PBP2a 246E variant), utilized in these studies are both β-lactamase-negative. This latter phenotype prevented testing these strains for differential penicillin-clavulanate vs. penicillin-alone MICs [13]. NaHCO_3_-responsive phenotypes were determined following MIC testing in cation-adjusted Mueller Hinton Broth (CA-MHB, Difco) containing 100 mM Tris (added to maintain a stable pH 7.3 ± 0.1 in the presence and absence of NaHCO_3_ as in our prior studies [6,7,9,10]); 44 mM NaHCO_3_ was employed as previously described, and represents tissue-level NaHCO_3_ concentrations [6,28,29]. MIC values for each strain in media with and without NaHCO_3_ are listed in Table S1. The specific PBP2a genotypes for each strain were determined by sequencing the PCR-amplified *mecA* product (GeneWiz LLC) [10]. Primers used for *mecA* amplification and sequencing are listed in Table S2.

### 4.2. Isolation of Purified PBP2a: Plasmid Construction 

Plasmids pET21b-*mecA*_246E_, pET21b-*mecA*_246G_, pET28b-*mecA*_246E_, and pET28b-*mecA*_246G_ were constructed using the In-Fusion Cloning kit (Takara Bio). *mecA* genes were amplified by PCR from genomic DNA of *S. aureus* strain COL and strain USA300 JE2 using the primer pairs A/B (for cloning into pET21b) or C/D (for cloning into pET28b). Plasmid backbones were generated by inverse PCR of pET21b and pET28b (Novagen) using primer pairs E/F and E/G, respectively. The resulting constructs were verified by Sanger DNA sequencing. Oligonucleotide primers (Table S2) were synthesized by Eurofin Genomics.

### 4.3. Isolation of Purified PBP2a: Overexpression and Purification of S. aureus PBP2a_246E_ and PBP2a_246G_

Expression cultures of *Escherichia coli* C43(DE3) transformed with the recombinant plasmids (pET21b containing *mecA* with a C-terminal His_6_-tag or pET28b containing *mecA* with an N-terminal His_6_-tag, respectively) were grown in 2 L of LB containing 100 µg/mL ampicillin for pET21b or 50 µg/mL kanamycin for pET28b at 37 °C with shaking. At an optical density (OD_600_) of 0.5, isopropyl β-D-thiogalactopyranoside (IPTG) was added to a concentration of 0.5 mM to induce expression of recombinant PBP2a. Expression cultures were cooled down to 18 °C and incubation was continued for 16 h. Cells were harvested by centrifugation (8000× *g*, 15 min, 4 °C) and resuspended in 20 mL of ice-cold lysis buffer (50 mM Tris-HCl, pH 7.5, 500 mM NaCl, 1% *v*/*v* Triton X-100). All subsequent steps were performed at 4 °C. Resuspended cells were incubated with 5 µg/mL RNase, 20 µg/mL DNase, and 0.5% *v*/*v N*-lauroylsarcosine for 1 h and disrupted by sonication (amplitude: 60%, pulse cycle 0.5). Cell debris was pelleted by centrifugation (48,000× *g*, 30 min, 4 °C) and the supernatant was added to 1 mL Ni-NTA-agarose (Macherey-Nagel). After gentle stirring for 1 h, the suspension was loaded to a column support. To remove weakly bound material, the column was washed with 10 mL wash buffer (50 mM Tris-HCl pH 7.5, 500 mM NaCl) supplemented with 10 mM imidazole and then with 10 mL wash buffer supplemented with 20 mM imidazole. His-tagged proteins were eluted 5 times with 500 μL wash buffer supplemented with 200 mM of imidazole and stored in 40% *v*/*v* glycerol at −20 °C. The purity of elution fractions was analyzed by SDS-PAGE (Figure S4) and concentrations were measured using Pierce™ BCA protein assay with BSA as standard.

### 4.4. Construction of JE2 and COL mecA 246-Residue Point Mutation Strains

To determine the contribution of the amino acid position at 246-residue (Glu/Gly) of the *mecA* gene in NaHCO_3_-responsive or non-responsive *S. aureus* strains, we constructed chromosomal point mutation of the *mecA* region in *S. aureus* strains JE2 (RBS: AGGAGT and 246G) and COL(*tet*^R^)(RBS:AGGAGG and 246E) using routine procedures as described [30]. To construct point mutation constructs, 3.2 kb DNA fragments were amplified that contained the intact *mecA* and *mecR* genes by PCR using primers flanked with BamHI sites at both ends and chromosomal DNA as the template of both JE2 and COL (Table S2). The DNA fragment was cloned into a temperature-sensitive shuttle vector pMAD (β-gal, *erm^R^*) [31], and then selected in *E. coli* IM08B [32] for the correct construct by digesting with BamHI of the isolated plasmid. To construct point mutation at the 246-residue position, site-specific mutagenesis was performed with pMAD constructs as the template and various mutagenized primers using a PCR-based method with *pfu* Taq-polymerase (Phusion, Thermo Scientific, Waltham, MA, USA). PCR products were treated with a KLD enzymes mix (NE Biolabs Inc., Ipswich, MA, USA) and transformed to *E. coli* IM08B for the selection on LB agar plates containing ampicillin (100 μg/)mL-X-Gal (40 μg/)mL. Final constructs were verified by restriction digestion and DNA sequencing. Authenticated desired respective constructs were introduced into JE2 and COL strains by electroporation and selected on erythromycin and X-Gal-containing plates (40 μg/)mL for blue colonies at 30 °C. Plasmid DNA was isolated and digested with BamHI for the authentication of the presence of DNA fragments in the respective constructs in the strains. The construction of chromosomal mutations in the respective strain by recombination or two-point cross-over was performed by routine procedure as described previously [30]. Briefly, two-point cross-over of the *mecA-mecR* region was performed by temperature shift by growing strains at 43 °C with erythromycin followed by 30 °C sub-culturing without any antibiotics. Cells were plated with and without erythromycin in the presence of X-Gal for selection and incubated at 37 °C. White/non-blue colonies were cross-streaked to select erm^S^ colonies for the potential two-point cross-over clones or mutants. The mutants were verified by chromosomal PCR and DNA sequencing of the PCR product for both RBS and 246-residue positions. Final clones were designated as ALC9165 (JE2, RBS: AGGAGT and 246E) and ALC9167 (COL, RBS:AGGAGG and 246G).

### 4.5. Bocillin-FL Binding to Purified PBP2a and β-Lactam Competition Studies

Bocillin-FL is a fluorescently labeled derivative of penicillin V [33], and was purchased from Thermo Fisher Scientific (Waltham, MA, USA). Based on in vitro MIC data performed with strain JE2, Bocillin-FL has similar antibacterial activity to “cold” penicillin G (Table S3). Based on numerous pilot studies, 50 µg/mL of N-terminal His_6_-tagged PBP2a_246E_ or PBP2a_246G_ were deemed as ideal, and utilized in all purified PBP2a binding studies. Purified proteins were incubated with 25 µM, 50 µM, or 100 µM Bocillin-FL for 20 min at 37 °C in a 20 µL final reaction volume and then incubated for 5 min at 95 °C with LDS loading dye. The reaction mixtures were then run on a NuPAGE^TM^ 4–12% Bis-Tris protein gel and imaged with an Azure c400 imager (Azure Biosystems). Fluorescence intensities of the gel images were analyzed and quantified by ImageJ software (version 1.52a). For competition studies, “cold” penicillin G or oxacillin (purchased from Sigma-Aldrich, St. Louis, MO, USA) was incubated with 50 µg/mL PBP2a at various concentrations (0, 0.5, 1, 5, 10, 50, 100, 500 µg/mL) for 10 min at 37 °C. Then, 50 µM Bocillin-FL was added to each reaction mixture, and incubated for an additional 20 min at 37 °C. A control comprising 50 µg/mL PBP2a_246G_ and 25 µM Bocillin-FL was run on each gel, to which the intensity of all other reactions on that gel were normalized. Following protein gel electrophoresis, imaging, and ImageJ analysis, the IC_50_ for each PBP2a variant and antibiotic was calculated by linear regression analysis in Excel.

Fold changes in Bocillin-FL fluorescence in the presence vs. absence of NaHCO_3_ were calculated by measuring the Bocillin-FL intensity in paired gels, with Bocillin-FL-PBP2a binding reactions performed with and without NaHCO_3_. The intensity observed in the presence of NaHCO_3_ was divided by the intensity observed in the absence of NaHCO_3_ for each Bocillin-FL concentration (25, 50, 100 µM) to determine the fold change in intensity for a given pair of gels at each Bocillin-FL concentration. The fold change in intensity for three gel pairs was used to determine the average fold change and calculate statistical significance (*p*-value) by a Student’s *t*-test for each Bocillin-FL concentration indicated for the two PBP2a protein variants.

For kinetics studies, 50 µg/mL PBP2a_246G_ and PBP2a_246E_ were incubated at 37 °C with 50 µM Bocillin-FL, with or without 44 mM NaHCO_3_. At each indicated time point, the reaction sample was removed, incubated for 5 min at 95 °C with LDS loading dye, and run on a NuPAGE^TM^ 4–12% Bis-Tris protein gel, as indicated previously. A control sample of 50 µg/mL PBP2a_246G_ and 25 µM Bocillin-FL, incubated for 20 min, was run with each gel as a normalization control. The time to reach “maximal binding” was assessed by identifying the first point at which Bocillin-FL intensity no longer increased or began to decrease for one or more subsequent time points, following extensive pilot studies. The “time to reach 50% maximal binding” was calculated by polynomial regression analysis in Excel.

### 4.6. Bocillin-FL Binding to Whole Cells by Flow Cytometry

JE2, COL, JE2 with COL PBP2a (246E), and COL with JE2 PBP2a (246G) cells were grown to mid-log phase (OD_600nm_ = 0.5) in CA-MHB Tris ± 44 mM NaHCO_3_ (pH = 7.3 ± 0.1) with half the MIC of oxacillin, as indicated in Table S1. Cells were then washed twice in phosphate-buffered saline (PBS) and adjusted to OD_600nm_ = 1.0; 500 µL of adjusted cells were incubated with 500 µL of 200 µM Bocillin-FL (final concentration = 100 µM), and incubated for 20 min at 37 °C. Bocillin-FL stained cells were washed three times in PBS, then diluted to a final concentration of ~5 × 10^7^ CFU/mL; 10,000 cells were then analyzed by flow cytometry on FACScalibur^®^ (Becton-Dickinson, Franklin Lakes, NJ, USA). The proportion of cells that bound Bocillin-FL was calculated using FlowJo software (version 10.8). In addition, the mean fluorescence intensity (MFI) of the cell population was calculated using data from the FL1-H channel, analyzed with FlowJo software and expressed as relative fluorescent units/cell population.

## 5. Conclusions

NaHCO_3_ differentially impacts binding affinities of the 246G and 246E PBP2a protein variants found in NaHCO_3_-responsive and -non-responsive strains, respectively. NaHCO_3_ enhances binding of the 246G variant to Bocillin-FL, as compared to the 246E variant, in both purified protein and whole-cell assays. Enhanced β-lactam binding to the 246G variant in the presence of NaHCO_3_ is correlated to increased susceptibility to β-lactams in the presence of NaHCO_3_ in responsive strains.

## Data Availability

Data are contained within the article (Figure 1C and Figure 4B–E). Additional datasets are available upon request.

## Supplementary Materials

The following are available online at https://www.mdpi.com/article/10.3390/antibiotics11040462/s1, Figure S1: “Cold” oxacillin and penicillin competition of Bocillin-FL binding to purified PBP2a variants (246G and 246E); Figure S2: Representative dot plot images of JE2 and COL PBP2a swap strains grown in CA-MHB Tris ± NaHCO_3_; Figure S3: Mean fluorescence intensity for JE2 and COL whole cells stained with Bocillin-FL; Figure S4: SDS-PAGE of purified PBP2a His_6_-tagged proteins; Table S1: Oxacillin minimum inhibitory concentrations (MICs) for JE2, COL, and their *mecA* swap derivative MRSA strains; Table S2: Oligonucleotides used for mecA sequencing, construction of His-tagged *mecA*_246E_ and *mecA*_246G_ plasmids, and construction of JE2 and COL *mecA* mutant variants; Table S3: MICs of penicillin G and Bocillin-FL in strain JE2.

## References

[1] Tong S.Y., Davis J.S., Eichenberger E., Holland T.L., Fowler V.G. (2015). *Staphylococcus aureus* infections: Epidemiology, pathophysiology, clinical manifestations, and management. Clin. Microbiol. Rev..

[2] Dien Bard J., Hindler J.A., Gold H.S., Limbago B. (2014). Rationale for eliminating *Staphylococcus* breakpoints for β-lactam agents other than penicillin, oxacillin or cefoxitin, and ceftaroline. Clin. Infect. Dis..

[3] Grundmann H., Aires-de-Sousa M., Boyce J., Tiemersma E. (2006). Emergence and resurgence of meticillin-resistant *Staphylococcus aureus* as a public-health threat. Lancet.

[4] Chambers H.F., Sachdeva M., Kennedy S. (1990). Binding affinity for penicillin-binding protein 2a correlates with in vivo activity of β-Lactam antibiotics against methicillin-resistant *Staphylococcus aureus*. J. Infect. Dis..

[5] Chambers H.F., Sachdeva M. (1990). Binding of β-lactam antibiotics to penicillin-binding proteins in methicillin-resistant *Staphylococcus aureus*. J. Infect. Dis..

[6] Ersoy S.C., Abdelhady W., Li L., Chambers H.F., Xiong Y.Q., Bayer A.S. (2019). Bicarbonate resensitization of methicillin-resistant *Staphylococcus aureus* to β-Lactam antibiotics. Antimicrob. Agents Chemother..

[7] Ersoy S.C., Otmishi M., Milan V.T., Li L., Pak Y., Mediavilla J., Chen L., Kreiswirth B., Chambers H.F., Proctor R.A. (2020). Scope and Predictive Genetic/Phenotypic Signatures of ‘Bicarbonate [NaHCO_3_]-Responsiveness’ and β-Lactam Sensitization Among Methicillin- Resistant *Staphylococcus aureus* (MRSA). Antimicrob. Agents Chemother..

[8] Ersoy S.C., Heithoff D.M., Barnes L.T., Tripp G.K., House J.K., Marth J.D., Smith J.W., Mahan M.J. (2017). Correcting a fundamental flaw in the paradigm for antimicrobial susceptibility testing. EBioMedicine.

[9] Ersoy S.C., Chambers H.F., Proctor R.A., Rosato A.E., Mishra N.N., Xiong Y.Q., Bayer A.S. (2021). Impact of bicarbonate on PBP2a production, maturation, and functionality in methicillin-resistant *Staphylococcus aureus*. Antimicrob. Agents Chemother..

[10] Ersoy S.C., Rose W.E., Patel R., Proctor R.A., Chambers H.F., Harrison E.M., Pak Y., Bayer A.S. (2021). A Combined Phenotypic-Genotypic Predictive Algorithm for In Vitro Detection of Bicarbonate: β-Lactam Sensitization among Methicillin-Resistant *Staphylococcus aureus* (MRSA). Antibiotics.

[11] Rose W.E., Bienvenida A.M., Xiong Y.Q., Chambers H.F., Bayer A.S., Ersoy S.C. (2020). Ability of bicarbonate supplementation to sensitize selected methicillin-resistant *Staphylococcus aureus* (MRSA) strains to β-Lactam antibiotics in an *ex vivo* simulated endocardial vegetation model. Antimicrob. Agents Chemother..

[12] Ersoy S.C., Hanson B.M., Proctor R.A., Arias C.A., Tran T.T., Chambers H.F., Bayer A.S. (2021). Impact of Bicarbonate-β-Lactam Exposures on Methicillin-Resistant *Staphylococcus aureus* (MRSA) Gene Expression in Bicarbonate-β-Lactam-Responsive vs. Non-Responsive Strains. Genes.

[13] Harrison E.M., Ba X., Coll F., Blane B., Restif O., Carvell H., Köser C.U., Jamrozy D., Reuter S., Lovering A. (2019). Genomic identification of cryptic susceptibility to penicillins and β-lactamase inhibitors in methicillin-resistant *Staphylococcus aureus*. Nat. Microbiol..

[14] Fey P.D., Endres J.L., Yajjala V.K., Widhelm T.J., Boissy R.J., Bose J.L., Bayles K.W. (2013). A genetic resource for rapid and comprehensive phenotype screening of nonessential *Staphylococcus aureus* genes. MBio.

[15] Jousselin A., Manzano C., Biette A., Reed P., Pinho M., Rosato A., Kelley W.L., Renzoni A. (2015). The *Staphylococcus aureus* chaperone PrsA is a new auxiliary factor of oxacillin resistance affecting penicillin-binding protein 2A. Antimicrob. Agents Chemother..

[16] Renzoni A., Kelley W.L., Rosato R.R., Martinez M.P., Roch M., Fatouraei M., Haeusser D.P., Margolin W., Fenn S., Turner R.D. (2017). Molecular bases determining daptomycin resistance-mediated resensitization to β-lactams (seesaw effect) in methicillin-resistant *Staphylococcus aureus*. Antimicrob. Agents Chemother..

[17] Lin M.H., Li C.C., Shu J.C., Chu H.W., Liu C.C., Wu C.C. (2018). Exoproteome profiling reveals the involvement of the foldase PrsA in the cell surface properties and pathogenesis of *Staphylococcus aureus*. Proteomics.

[18] de Carvalho C.C., Taglialegna A., Rosato A.E. (2022). Impact of PrsA on membrane lipid composition during daptomycin-resistance-mediated β-lactam sensitization in clinical MRSA strains. J. Antimicrob. Chemother..

[19] Fan S.-H., Ebner P., Reichert S., Hertlein T., Zabel S., Lankapalli A.K., Nieselt K., Ohlsen K., Götz F. (2019). MpsAB is important for *Staphylococcus aureus* virulence and growth at atmospheric CO_2_ levels. Nat. Commun..

[20] Otero L.H., Rojas-Altuve A., Llarrull L.I., Carrasco-López C., Kumarasiri M., Lastochkin E., Fishovitz J., Dawley M., Hesek D., Lee M. (2013). How allosteric control of *Staphylococcus aureus* penicillin binding protein 2a enables methicillin resistance and physiological function. Proc. Natl. Acad. Sci. USA.

[21] Muñoz V., Serrano L. (1995). Elucidating the Folding Problem of Helical Peptides using Empirical Parameters. III> Temperature and pH Dependence. J. Mol. Biol..

[22] Roch M., Lelong E., Panasenko O.O., Sierra R., Renzoni A., Kelley W.L. (2019). Thermosensitive PBP2a requires extracellular folding factors PrsA and HtrA1 for *Staphylococcus aureus* MRSA β-lactam resistance. Commun. Biol..

[23] Farha M.A., French S., Stokes J.M., Brown E.D. (2017). Bicarbonate alters bacterial susceptibility to antibiotics by targeting the proton motive force. ACS Infect. Dis..

[24] Mahasenan K.V., Molina R., Bouley R., Batuecas M.T., Fisher J.F., Hermoso J.A., Chang M., Mobashery S. (2017). Conformational dynamics in penicillin-binding protein 2a of methicillin-resistant *Staphylococcus aureus*, allosteric communication network and enablement of catalysis. J. Am. Chem. Soc..

[25] Fuda C., Hesek D., Lee M., Morio K.-I., Nowak T., Mobashery S. (2005). Activation for Catalysis of Penicillin-Binding Protein 2a from Methicillin-Resistant Staphylococcus a Ureus by Bacterial Cell Wall. J. Am. Chem. Soc..

[26] Meisel J.E., Fisher J.F., Chang M., Mobashery S. (2018). Allosteric inhibition of bacterial targets: An opportunity for discovery of novel antibacterial classes. Antibacterials.

[27] Fishovitz J., Rojas-Altuve A., Otero L.H., Dawley M., Carrasco-López C., Chang M., Hermoso J.A., Mobashery S. (2014). Disruption of allosteric response as an unprecedented mechanism of resistance to antibiotics. J. Am. Chem. Soc..

[28] Cockerill F.R. (2012). Methods for Dilution Antimicrobial Susceptibility Tests for Bacteria That Grow Aerobically: Approved Standard.

[29] Weinstein M.P., Patel J.B., Campeau S., Eliopoulos G.M., Galas M.F., Humphries R.M., Jenkins S.G., Lewis J.S., Limbago B., Mathers A.J. (2018). Performance Standards for Antimicrobial Susceptibility Testing.

[30] Kim S., Reyes D., Beaume M., Francois P., Cheung A. (2014). Contribution of teg49 small RNA in the 5′ upstream transcriptional region of sarA to virulence in *Staphylococcus aureus*. Infect. Immun..

[31] Arnaud M., Chastanet A., Débarbouillé M. (2004). New vector for efficient allelic replacement in naturally nontransformable, low-GC-content, gram-positive bacteria. Appl. Environ. Microbiol..

[32] Monk I.R., Tree J.J., Howden B.P., Stinear T.P., Foster T.J. (2015). Complete bypass of restriction systems for major *Staphylococcus aureus* lineages. MBio.

[33] Zhao G., Meier T.I., Kahl S.D., Gee K.R., Blaszczak L.C. (1999). Bocillin-FL, a sensitive commercially available reagent for detection of penicillin-binding proteins. Antimicrob. Agents Chemother..

