# Supplementary material for: Impacts of NaHCO3 on β-Lactam Binding to PBP2a Protein Variants Associated with the NaHCO3-Responsive versus NaHCO3-Non-Responsive Phenotypes
Source: Antibiotics (Basel). 2022 Mar 30;11(4):462. doi: 10.3390/antibiotics11040462 (PMC9028190; doi:10.3390/antibiotics11040462)
Supplement: Supplementary file 1 [file antibiotics-11-00462-s001.zip › antibiotics-1639634-supplementary.pdf]

Supplementary Materials

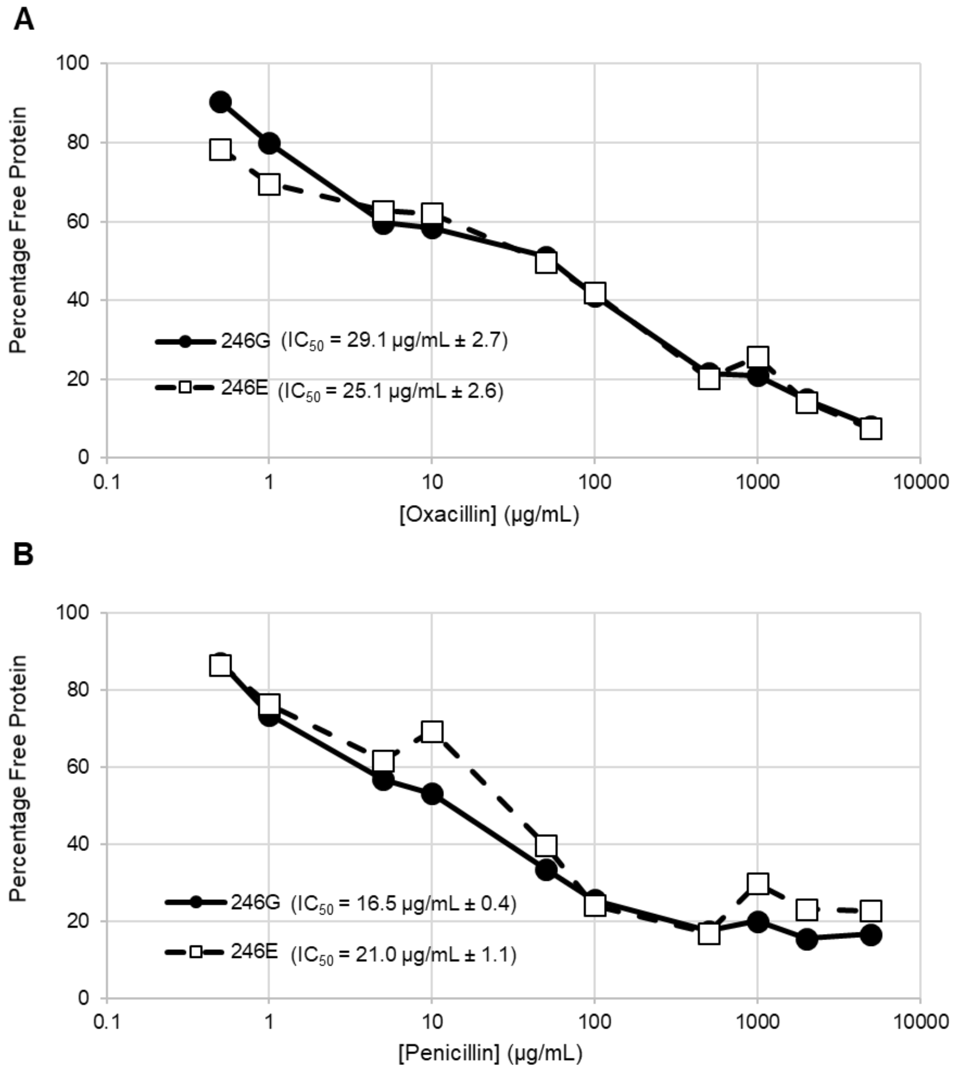

Figure S1. ‘Cold’ oxacillin (**A**) and penicillin (**B**) competition of Bocillin-FL binding to purified PBP2a variants (246G and 246E variants).

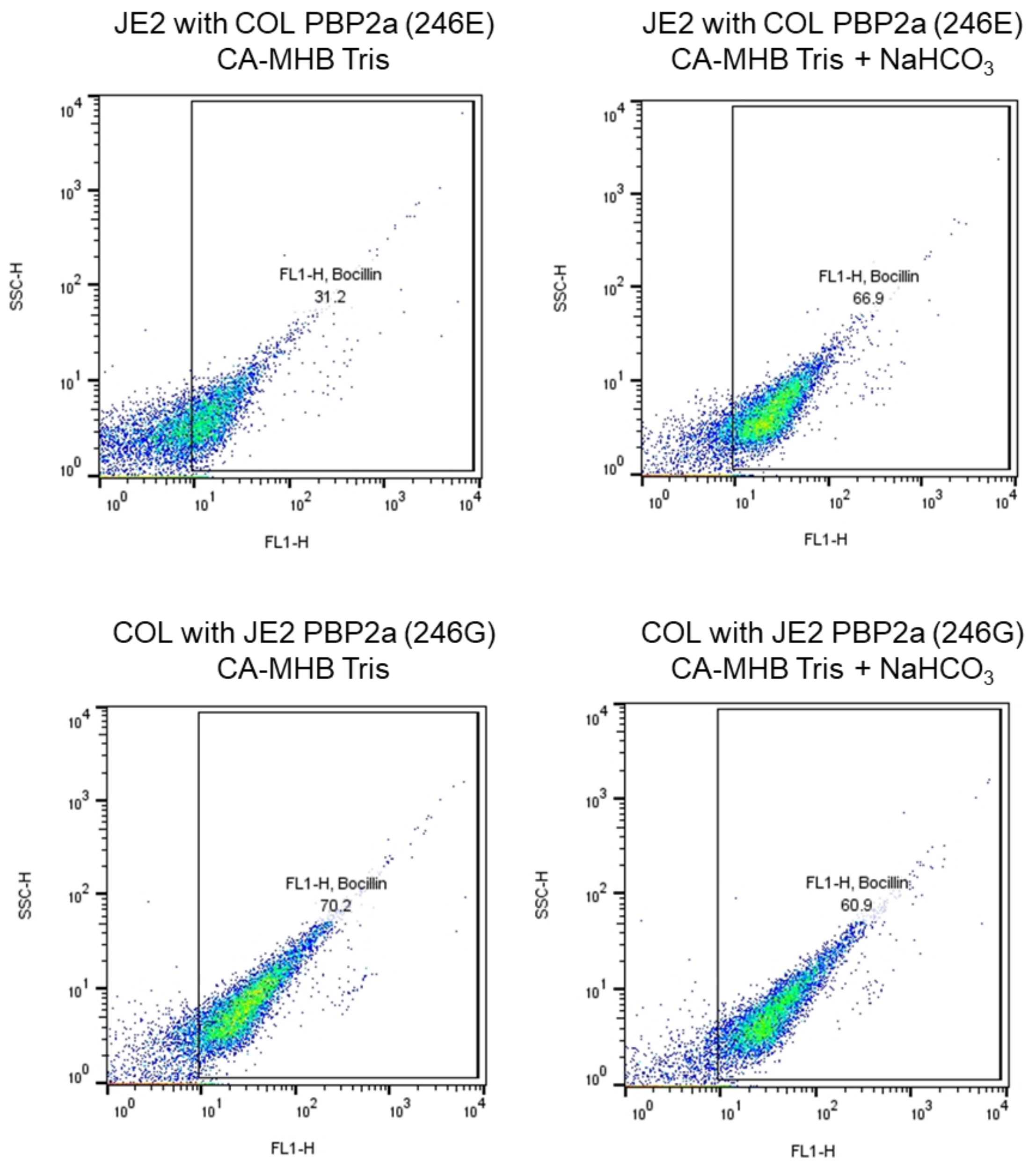

Figure S2. Representative dot plot images of JE2 and COL PBP2a swap strains grown in CA-MHB Tris ± $NaHCO_3$.

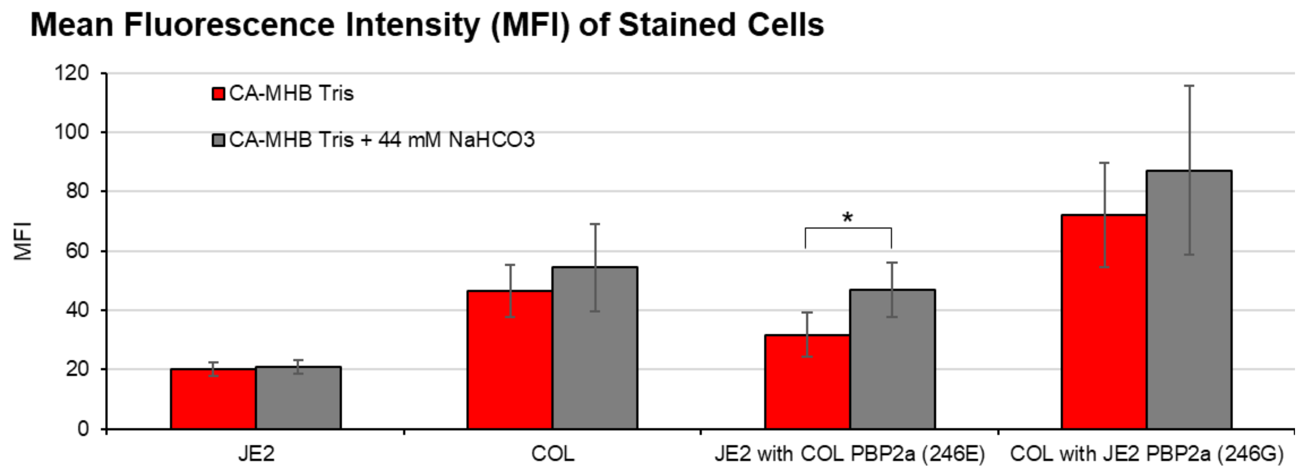

Figure S3. Mean fluorescence intensity of JE2 and COL whole cells stained with Bocillin-FL, following growth in CA-MHB Tris media, with and without $NaHCO_3$. Statistics were calculated by a Student's *t-test* (*P < 0.05).

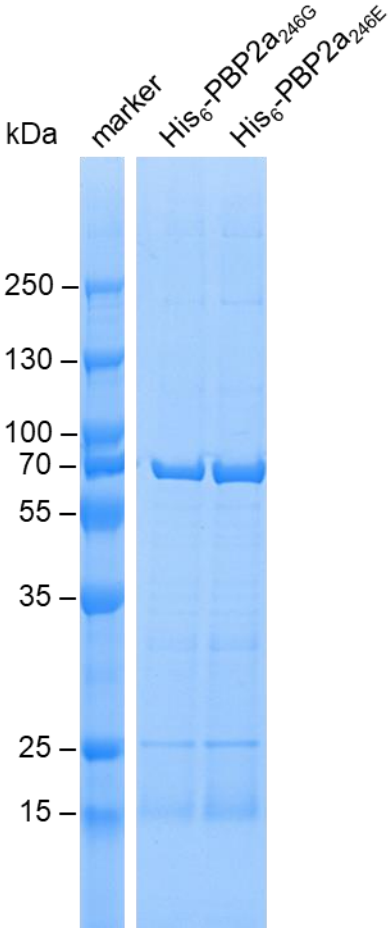

Figure S4. SDS-PAGE of purified PBP2a His_6-tagged proteins.

Table S1. Oxacillin minimum inhibitory concentrations (MICs) for JE2, COL, and their *mecA* swap mutant MRSA strains. MIC determinations were performed in the presence of 2% NaCl.

	Oxacillin MIC (µg/mL)	
Strain	Ca-MHB Tris	Ca-MHB Tris 44 mM $NaHCO_3$
JE2	64	1
COL	512	512
JE2 with COL PBP2a (246E)	64	2
COL with JE2 PBP2a (246G)	512	512

Table S2. Oligonucleotides used for mecA sequencing, construction of His-tagged *mecA*$_{246E}$ and *mecA*$_{246G}$ plasmids, and construction of JE2 and COL *mecA* mutant variants.

Primer designation	Name	Sequence (5‘ to 3‘)	Application
None	*mecA* F0	ACCGAAGAAGTCGTGTCAGA	PCR amplification (for sequencing)
None	*mecA* R2	ACGTTGTAACCACCCCAAGA	PCR amplification (for sequencing)
None	*mecA* F1	ATTAACGTGGAGACGAGCACT	Sequencing
None	*mecA* F2	CGGTAACATTGATCGCAACG	Sequencing
None	*mecA* R3	TCGTCAACGATTGTGACACG	Sequencing
None	*mecA* R4	CGAAGGTATCATCTTGTACCCA	Sequencing
A	fwd_mecA-Cterm	GATATACATATGGCTATGAAAAAGATAAAAATTGTTCCA	PBP2a protein purification
B	rev_mecA-Cterm	GTGGTGGTGGTGCTCTTCATCTATATCGTATTTTTTATT	PBP2a protein purification
C	fwd_mecA-Nterm	GGCAGCCATATGGCTATGAAAAAGATAAAAATTGTTCCA	PBP2a protein purification
D	rev_mecA-Nterm	GTGGTGGTGGTGCTCTTATTCATCTATATCGTATTTTTT	PBP2a protein purification
E	fwd_pET21b/pET28b	GAGCACCACCACCACCAC	PBP2a protein purification
F	rev_pET21b	AGCCATATGTATATCTCCTTC	PBP2a protein purification
G	rev_pET28b	AGCCATATGGCTGCCGCG	PBP2a protein purification

None	731	AAGAGAAGGATCCATCGTTACGGATTGCTTCACTGTT	Cloning
None	732	AAGAGAAGGATCCTTAACTGTTTTAGTTAACTCATGT	Cloning
None	733	AACCAAATCTTATGTGACATAATATTT	PCR verification
None	734	GTAATCTGGAACTTGTTGAGCAGA	PCR verification
None	745 (*mecA* 246G fwd)	TTC CTA GAG GAT AGT TAC GAC TTT CTG	Mutagenesis AA246
None	746 (*mecA* 246E fwd)	TTT CTA GAG GAT AGT TAC GAC TTT CTG	Mutagenesis AA246
None	747 (*mecA* 246 rev)	AAG CGA CTT CAC ATC TAT TAG GTT A	Mutagenesis AA246

Table S3. JE2 MICs (µg/mL) for Penicillin G and Bocillin-FL (Penicillin V conjugated to FL).

Antibiotics	Ca-MHB
PEN G	2
Bocillin -FL	2
